# An investigation of the association between religious coping, fatigue, anxiety and depressive symptoms during the COVID-19 pandemic in Morocco: a web-based cross-sectional survey

**DOI:** 10.1186/s12888-021-03271-6

**Published:** 2021-05-22

**Authors:** Btissame Zarrouq, Nivine Abbas, Jaouad El Hilaly, Achraf El Asri, Samira Abbouyi, Majid Omari, Hicham Malki, Samira Bouazza, Salma Ghofrane Moutawakkil, Karima Halim, Mohammed Elamine Ragala

**Affiliations:** 1 Laboratory of Epidemiology and Research in Health Sciences, Faculty of Medicine and Pharmacy, Sidi Mohamed Ben Abdellah University, Fez, Morocco; 2 Teachers Training College (Ecole Normale Superieure), Department of Biology and Geology, Sidi Mohamed Ben Abdellah University, Fez, Morocco; 3 Public Health Department, Faculty of Health Sciences, University of Balamand, Balamand, El-Koura Lebanon; 4 Laboratory of Pedagogical and Didactic Engineering of Sciences and Mathematics, Regional Center of Education and Training (CRMEF) of Fez, Fez, Morocco; 5 Laboratory of Natural Substances, Pharmacology, Environment, Modeling, Health & Quality of Life, Faculty of Sciences Dhar El Mahraz, Sidi Mohamed Ben Abdellah University, Fez, Morocco; 6 Teachers Training College (Ecole Normale Superieure), Department of Human and Social Sciences - Education Sciences, Sidi Mohamed Ben Abdellah University, Fez, Morocco

**Keywords:** Depression, Anxiety, Fatigue, Religious coping, COVID-19, Quarantine, Morocco

## Abstract

**Background:**

The Coronavirus Disease 2019 (COVID-19) pandemic has triggered fear and distress among the public, thus potentiating the incidence rate of anxiety and depression. This study aims to investigate the psychological effect of quarantine on persons living in Morocco when the first COVID-19 cases were identified. The associations between anxiety, depression symptoms, and their predictors (sociodemographics, fatigue, and religious coping) were examined.

**Methods:**

A web-based cross-sectional survey, with a total of 1435 participants (≥18 years) recruited anonymously, was conducted during the COVID-19 pandemic (from 3 to 30 April 2020). A structured questionnaire was used to assess psychosocial factors, COVID-19 epidemic-related factors, and religious coping. Religious coping, fatigue, and depression, and anxiety were measured by Brief Religious Coping Scale (Brief RCOPE), Chalder Fatigue Scale (CFS), and Hospital Anxiety and Depression Scale (HADS), respectively. A generalized linear model (logistic regression) was used to determine the predictive factors of depression and anxiety.

**Results:**

The prevalence of anxiety and depression was 43.0% (*n* = 621) and 53.0% (*n* = 766), respectively. Both were associated with female gender, household income decline, tracking COVID-19 news, and fear to contract COVID-19 (aOR = 1.36 to 2.85). Additionally, 32.0% (*n* = 453) and 26.0% (*n* = 372) reported severe physical fatigue, and mental fatigue, respectively. Both latter factors were significantly and positively associated with depression as well as with anxiety. Depressive and anxious patients used more negative religious coping, while positive religious coping was slightly associated with depression.

**Conclusion:**

In this online survey of the general population in Morocco, anxiety and depressive symptoms are prevalent during the COVID-19 pandemic. Pandemic and psychosocial factors, such as female gender, income decline, infection fears, massive COVID-19 news exposure, negative religious coping, and fatigue were associated with increased risk of depression and anxiety symptoms. Psychosocial and financial support should be provided to the quarantined population.

**Supplementary Information:**

The online version contains supplementary material available at 10.1186/s12888-021-03271-6.

## Background

Coronavirus disease 2019 (COVID-19) was first reported in China. Shortly thereafter, the disease spread all over the world to became a global pandemic [1, 2]. Since the severe acute respiratory syndrome outbreak in 2003, COVID − 19 is deemed to be highly contagious; it has affected 219 countries in record time [3]**.** On March 2nd, 2020, the first coronavirus case was officially confirmed in Morocco [4]. Up to April 10th 2021, 500,948 have been diagnosed as COVID-19 positive, of which 8885 were fatal [5]. Moroccan authorities, for the sake of control and prevention, have implemented urgently a package of restrictive measures, such as isolation of suspected and diagnosed cases, cancelling parties, extending holidays, closing schools, and complying with stay-at-home orders [6].

A couple of studies have reported that the outbreaks of fatal infectious diseases impact negatively people’s mental health and well-being [7]. Indeed, concerns about the mental health and psychological adjustment of the public have been arising due to the COVID-19’s quick outbreaks and high mortality. Accordingly, a couple of works have reported significant psychosocial impacts of the COVID-19 quarantine on mental and physical health, such as post-traumatic stress symptoms, depression, anxiety, fear, and confusion among general population and medical workers as well. The severity of psychological disorders varies depending on sociodemographic factors, such as age, gender, area of living, social media, income, social support, religiosity, having relatives infected or died from COVID-19 [8, 9], guilt about the effects of contagion [10], inadequate or fake information especially from unauthorized sources or media [11, 12], uncertainty and unpredictability around COVID-19 [13], quarantine duration, frustration, fear of losing friends and family [11], loneliness, boredom, inadequate supplies and health equipment, financial loss, and stigma [10, 14]. All these stressors increase anxiety and depression in the quarantined population. Moreover, studies showed that prolonged feelings of distress and worry can increase levels of fatigue [15].

On the other hand, people experiencing adverse life events tend to take religion as a source of comfort [16], and adopt more religious coping [17–20]. Religious coping can be defined as the way individuals use cognitive or behavioral strategies based on religious beliefs or practices to cope with stress and life problems [21]. This can include reading holy scriptures, seeking counsel from religious leaders, and reducing unpleasant thoughts using religious means [19]. Literature has argued that religiosity can be seen as a framework of meaning-making that is associated with decreased psychological distress and thus a powerful resource for mental health [22, 23]. Positive religious coping provides a source of social support [24]. The majority of the Moroccan population is Muslim. Islamic beliefs state that every event, whether positive or negative, has a purpose, and spiritual growth requires enormous patience and commitment [17, 25]. Literature has mentioned that the Islamic religion plays an important role in protecting people from difficult mental health outcomes including attempts to suicide [19].

Although quarantine measures are successful in reducing or terminating the COVID-19 outbreak in different areas of the world, the adverse effects of quarantine have not previously been studied systematically. Literature has stated that there is not enough data available about the effect of the COVID − 19 pandemic on mental health problems among the general population [10, 26].

Up to our knowledge, information about the prevalence of depression, anxiety, and fatigue of Moroccan citizens, and their association with religious coping during the COVID-19 outbreak have not been studied yet. To this end, we conducted an online investigation during the first wave of COVID-19 pandemic to investigate the psychological effect of quarantine on people’s psychological symptoms, mainly depression, anxiety, and their associations with the sociodemographic, fatigue and religious coping factors.

## Methods

### Participants

In this study, the snowball method was adopted to carry out the distribution of questionnaires due to the difficulty of reaching people during the COVID-19 total lockdown. Ten initial seeds were chosen to ensure a broad representation of age, gender, and educational level. These seeds sent the questionnaire link, via WhatsApp groups, Facebook groups, and other social media, to other participants who must be Moroccan citizens, aged more than 18 years, and so it goes on. The participants were directed to the survey link through Google form and responded only once to the questionnaire voluntarily and anonymously. The snowball sampling process continued between 3 to 30 April 2020 until sufficient sample size was reached. The online snowball sampling does not allow an assessment of the response rate. Apart from the seed vertices, the researchers do not have control over which vertices are included in the sample.

All respondents had provided electronic informed consent before starting the investigation. Ethical approval was obtained from the hospital-university ethics committee of Sidi Mohamed Ben Abdellah University (N°09/20). Researchers had ensured that the internationally recognized ethical principles for research involving human subjects were respected throughout this research, and all methods were carried out per relevant guidelines and regulations.

### Measures

This online survey was implemented by using a structured questionnaire composed of 56 multiple-choice questions (Supplementary file). It took approximately 15 min to be completed.

### Sociodemographics

Sociodemographic data including participants’ age, gender, education, marital status, parental status, employment status, and living arrangements were monitored. Participants were classified according to their level of education (higher, secondary, and primary education), age stages, employment status (private sector, public sector, self-employment), and living arrangement (living alone or with others).

### COVID-19-related information sources, knowledge and worry about COVID-19

The frequency with which the respondents received COVID-19-related information was explored, the COVID-19 Knowledge (route of transmission, the availability of medicines, the number of infected cases and death, advice on prevention of the COVID-19) was also reported. Besides, the levels of adherence to quarantine and infection control directives (always washing hands, wearing a mask, and covering mouth when coughing and sneezing) were investigated. The participants were asked to report their concern about COVID-19 (COVID-19 infection of relatives and acquaintances, fear of death from this disease, chronic disease, and income decline).

### Anxiety, depression, fatigue, and religiosity

The psychological impact of COVID-19 and quarantine was evaluated by the Hospital Anxiety and Depression Scale (HADS) [27]. It includes 14 items assessing anxiety (7 items) and depression (7 items), which are rated on a 4-point Likert type (from 0 to 3). The scores in each subscale are computed by summing the corresponding items, with maximum scores of 21 for each subscale. Zigmond and Snaith have advocated cut-offs between 8 and 10 for possible cases, and scores of 11 or more for definite cases of depression and anxiety [27]. Choosing the more conventional cut-off point of 11 would have missed out a significant proportion of people with anxiety and depressive symptoms. In this study, the cut-off of 8 was chosen for the Arabic version of HADS scales. Participants who obtained a score > 7 on HADS-Depression subscale (HADS-D) and /or HADS-Anxiety subscale (HADS-A) were considered as having symptoms suggestive of depression and/ or anxiety, respectively [28]. The HADS scale was chosen because it is easy and brief, and measures both anxiety and depressive symptoms with good reliability and validity in general population [28]. However, other scales, such as the Center for Epidemiologic Studies Depression Scale (CESD), and the Patient Health Questionnaire-9 (PHQ-9) measure only depressive symptoms. The former one is too long, while the latter one has a sensitive and unsuitable item for a general population during the stressful time of a pandemic [29]. The reliability and validity of the Arabic version of the HADS scale were previously reported [30]. The internal consistency of the HADS scales in the current study is acceptable; and this is true for both anxiety (α-Cronbach = 0.88) and depression (α-Cronbach = 0.76).

Physical and mental fatigue was assessed by the Chalder Fatigue Scale **(**CFS) [31], and a validated Arabic version was used [32]. It consists of 11 items and two dimensions: physical fatigue (7 items) and mental fatigue (4 items). Participants were asked to mention their experienced symptoms of mental and physical fatigue in the last 2 weeks. In the current work, the internal consistency of the scale is significant; the Cronbach’s alpha of CFS-physical fatigue was acceptable (α-Cronbach = 0.82), whereas that of CFS-mental fatigue was questionable (α-Cronbach = 0.61).

Religious coping was evaluated with the Brief Religious Coping Scale (Brief RCOPE). This scale is a 14-item measure of religious coping with crisis, trauma, and transition [33]. Brief RCOPE is the most commonly used measure of religious coping in the literature [34]. This scale is divided into two subscales: positive and negative religious coping. The positive religious coping subscale (P RECOPE) of the Brief RCOPE taps into a sense of connectedness with a transcendent force, a secure relationship with a caring God, and a belief that life has a greater benevolent meaning. The negative religious coping subscale (N RECOPE) of the Brief RCOPE is characterized by signs of spiritual tension, conflict and struggle with God and others, spiritual questioning and doubting, and interpersonal religious discontent [33]. Both Brief RCOPE subscales range from 7 (low) to 28 (high). A four-point Likert scale was used with this scale, and the response options were from 1 (strongly disagree) to 4 (strongly agree) [17]. The reliability and validity of the Arabic version of Brief RCOPE were established in a previous study [17]. P RECOPE and N RECOPE had high internal consistency in this study (Cronbach’s α= = 0.85, 0.80, respectively).

### Statistical analyses

The sample size was calculated based on the estimated rate of depressive symptoms in the general population. Epidemiological data reported the lifetime prevalence of depressive symptoms in the Moroccan population as 26.5% [35], and the prevalence of anxiety as 25.5% [36]. Considering the likelihood of increased rates of depressive and anxiety symptoms during pandemics, we hypothesized a rate of 36.5% for depressive and anxiety symptoms in the general population. With a significance level set at .05, and an absolute error of 0.1%, we estimated a minimum sample size of 1435 for the current study.

All statistics were done using R software v.4.0.4 (http://www.r-project.org) implemented with gtsummary, Raven, Psych, EpiR, and tidyverse packages. Before conducting the study, a pilot test was conducted on 40 participants to assess the right connotation of each question. The results of the pilot study were not included in the analysis. The internal consistency was assessed using split-half reliability by splitting the test in half based on odd-numbered and even-numbered questions; each half of the test was administered to the same individuals (*N* = 150). The correlation between the scores for both halves was determined. Besides, the factorial structure of the 3 scales (HADS, CFS, RCOPE) were examined on this first sample (*N* = 150) using Exploratory Factor Analysis (EFA) (data not shown).

Descriptive statistics were used to examine the characteristics of the study population. Categorical variables were depicted in percentages and frequencies. Continuous variables were presented in means and standard deviation. The number of subjects with depression (HADS-depression subscale scores ≥8) and anxiety (HADS anxiety subscale scores ≥8) was computed. Chi-squared statistics were used to evaluate the associations between sociodemographic characteristics, depression, and anxiety. The assumptions for the general linear model were tested; and since they were not fulfilled, univariate and multivariate logistic regression, as a generalized linear model (GLM), were performed to generate the outcome of the dependent variables (depression and anxiety) from an exponential family of distribution. It was used to reveal the variables that are independently associated with depressive and anxious symptoms, test our previous hypothesis that COVID-19 related independent variables would account for a significant amount of depression and anxiety variance. Pseudo R-squared values were calculated using the Nagelkerke’s method [37]. The associations were presented using odds ratios (ORs) and their 95% confidence. The level of significance was set as *p* < 0.05 (two-tailed).

## Results

Out of 1530 questionnaires collected, 1435 (93.7%) questionnaires were fully completed by Moroccan citizens aged more than18 years. The usage of an online snowball sampling does not permit assessing the survey response rate, as there is no way to determine exactly how many people received the link and did not respond to it.

Participant’s average age was 32.2 ± 10.5 years (age ranged from 18 and 74 years). More than half of the participants were male (*n* = 811, 57.0%), not married (*n* = 827, 58.0%), and employed in the public sector (*n* = 769, 54.0%). Most respondents were well-educated (*n* = 1332, 93.0%), and had no chronic disease (*n* = 1312, 91.0%) (Table 1).

**Table 1:** Descriptive statistics of socio-demographic and psychological variables

Variable	*N* = 1435	
	*n (%)*	*Mean (SD)*
Age (Years)		32.2 (10.5)
Age stages (Years)		
≤ 45	1234 (86.0)	
> 45	201 (14.0)	
Gender		
Female	624 (43.0)	
Male	811 (57.0)	
Marital status		
Married	608 (42.0)	
Not married	827 (58.0)	
Status as a parent		
Having children	469 (33.0)	
No children	966 (67.0)	
Education		
Higher education	1332 (93.0)	
Primary or secondary education	103 (7.0)	
Living arrangements		
Alone	122 (8.5)	
Not alone	1313 (91.5)	
Employment status		
Employed in the private sector	108 (7.1)	
Employed in the public sector	769 (54.0)	
Retired	30 (2.1)	
Self-employed	75 (5.2)	
Student	373 (26.0)	
Unemployed	80 (5.6)	
Household income decline		
Yes	403 (28.0)	
No	1032 (72.0)	
Life satisfaction during the COVID-19 pandemic		
Satisfied	1124 (78.0)	
Not satisfied	311 (22.0)	
Looked up regular news		
Regularly	1301 (91.0)	
Seldom	134 (9.0)	
Compliance with protective measures		
High	979 (68.0)	
Low	456 (32.0)	
Compliance with quarantine measures		
High	1213 (85.0)	
Low	222 (15.0)	
Chronic illness		
Yes	123 (9.0)	
No	1312 (91.0)	
Fear of COVID-19		
High	454 (32.0)	
Low	981 (68.0)	
Fear of dying from COVID-19		
High	100 (7.0)	
Low	1335 (93.0)	
Know someone died from COVID-19		
Yes	34 (2.0)	
No	1401 (98.0)	
Depression		
Positive	766 (53.0)	10.2 (2.8)
Negative	669 (47.0)	3.6 (1.8)
Anxiety		
Positive	621 (43.0)	10.6 (3.4)
Negative	814 (57.0)	3.1 (1.9)
Physical fatigue		
Severe fatigue	453 (32.0)	5.2 (1.1)
No fatigue	982 (68.0)	1.0 (1.1)
Mental fatigue		
Severe fatigue	372 (26.0)	2.3 (0.5)
No fatigue	1063 (74.0)	0.2 (0.3)
Positive religious coping		17.8 (4.5)
Negative religious coping		9.3 (3.2)

*SD* Standard deviation

Regarding knowledge about COVID-19, nearly all respondents (*n* = 1301, 91.0%) showed a need to be regularly updated with the latest information about COVID 19 (route of transmission, the availability of medicines, the number of infected cases and death, and advice on COVID-19 control and prevention). Most participants expressed a high level of compliance with quarantine and protective measures (*n* = 1213, 85.0%; and *n* = 979, 68.0% respectively), whereas 32.0% (*n* = 454) of participants expressed a high level of fear to contract COVID-19.

Among the 1435 participants, 53.0% (*n* = 766) subjects were found to be depressed, 43.0% (*n* = 621) were anxious, 32.0% (*n* = 453) were suffering from severe physical fatigue, and 26.0% (*n* = 372) were suffering from mental fatigue. Concerning religious coping, the participants’ mean score of P RCOPE was high (M = 17.8, SD = 4.5), while the mean score of N RCOPE was low (M = 9.3, SD = 3.2) (both Brief RCOPE subscales range from 7 (low) to 28 (high)).

The association between depression, anxiety and sociodemographic independent variables was assessed by the Chi-square test. As shown in Table 2, the younger age (≤ 45 years) (depression OR = 1.81, *p* < 0.001; anxiety OR = 1.67, *p* < 0.001), female gender (depression OR = 1.63, *p* < 0.001; anxiety OR = 2.28, *p* < 0.001), household income decline (depression OR = 1.63, *p* < 0.001; anxiety OR = 1.49, *p* < 0.001), tracking regularly COVID-19 news (depression OR = 1.79, *p* < 0. 01; anxiety OR = 1.97, *p* < 0.001), fear of contracting COVID-19 (depression OR = 2.24, < 0.001; anxiety OR = 3.28, *p* < 0.001), and fear of dying from COVID-19 (depression OR = 6.46, *p* < 0.001; anxiety OR = 11.03, *p* < 0.001) showed significant association with both anxiety and depression (HADS scores of 8 and above for both anxiety and depression).

**Table 2:** Analysis of the association between demographic characteristics with depression and anxiety (based on HADS scores) among the study subjects using Chi-square test

Variable	Depression		χ2	OR	95% CI	Anxiety		χ2	OR	95% CI
	Positive (n)	Negative (n)				Positive (n)	Negative (n)			
Age stages (years)										
≤ 45	684	550	14.29***	1.81***	1.31–2.50	555	679	9.89***	1.67**	1.20–2.32
> 45	82	119				66	135			
Gender										
Female	376	248	20.49***	1.63***	1.31–2.04	341	283	57.35***	2.28***	1.81–2.85
Male	390	421				280	531			
Marital status										
Married	307	301	3.333	0.81	0.65–1.01	259	349	0.15	0.95	0.76–1.19
Not married	459	368				362	465			
Status as a parent										
Having children	226	243	7.24**	0.73**	0.58–0.91	195	274	0.72	0.90	0.71–1.13
No children	540	426				426	540			
Education										
Higher education	711	621	7.46	1.00	0.66–1.53	574	758	0.16	0.90	0.59–1.38
Primary or secondary education	55	48				47	56			
Living arrangements										
Alone	67	55	0.07	1.07	0.72–1.58	48	74	0.67	0.83	0.56–1.25
Not alone	699	614				573	740			
Household income decline										
Yes	250	153	16.39***	1.63***	1.28–2.08	203	200	11.10***	1.49***	1.17–1.88
No	516	516				418	614			
Life satisfaction during the COVID-19 pandemic										
Satisfied	529	595	81.96***	0.28***	0.21–0.37	420	704	72.66***	0.33***	0.25–0.43
Not satisfied	237	74				201	110			
Looked up regular news										
Regularly	712	589	9.59**	1.79**	1.23–2.63	582	719	11.46***	1.97***	1.31–3.03
Seldom	54	80				39	95			
Compliance with protective measures										
High	505	474	3.78	0.79	0.63–1.00	429	550	0.31	1.07	0.85–1.35
Low	261	195				192	264			
Compliance with quarantine measures										
High	651	562	0.19	1.07	0.80–1.44	526	687	0.01	1.02	0.75–1.38
Low	115	107				95	127			
Chronic illness										
Yes	76	47	3.46	1.45	0.98–2.17	71	52	10.81**	1.89***	1.28–2.81
No	690	622				550	762			
Fear of COVID-19										
High	303	151	46.85***	2.24***	1.75–2.85	286	168	104.04***	3.28***	2.56–4.16
Low	463	518				335	646			
Fear of dying from COVID-19,										
High	87	13	47.38***	6.46***	3.57–12.5	88	12	85.64***	11.03***	5.88–25.0
Low	679	656				533	802			
Know someone died from COVID-19										
Yes	15	19	0.85	0.68	0.32–1.43	15	19	3.37	1.03	0.49–2.17
No	751	650				606	795			

*χ2* Chi-square test, *OR* odds ratio, *CI* Confidence interval

*p < 0.05, **p < 0.01, ***p < 0.001

Participants with a chronic illness had 1.89 (*p* < 0.001) higher odds of having anxiety symptoms. Meanwhile, respondents who were satisfied with their life during the COVID-19 pandemic were less likely to be depressed or anxious (OR = 0.28, 95% CI: 0.21–0.37) and (OR = 0.33, 95% CI: 0.25–0.43) for depression and anxiety, respectively).

The correlations between the studied scales and subscales were moderate to strong and generally significant. CFS-Physical and mental fatigue, HADS-A, and HADS-D were positively correlated with negative religious coping and inversely correlated with positive religious coping (Table 3).

**Table 3:** Correlations between Chalder Fatigue scale (CFS), Hospital Anxiety and Depression Scale (HADS), and Brief Religious Coping Scale (Brief RCOPE)

	Mean	SD	α	2	3	4	5	6	7	8	9
1- CFS - Physical fatigue	2.34	2.27	0.82	0.58***	0.91***	0.56***	0.53***	0.59***	−0.02	0.12***	0.05
2- CFS - Mental fatigue	0.75	1.04	0.61	–	0.51***	0.48***	0.48***	0.52***	−0.03	0.14***	0.06*
3- CFS - Total	3.09	2.52	0.60		–	0.49***	0.45***	0.51***	−0.02	0.12***	0.05
4- HADS - Depression	7.19	4.10	0.76			–	0.69***	0.91***	−0.04	0.16***	0.05*
5- HADS - Anxiety	6.38	4.62	0.88				–	0.93***	0.09***	0.23***	0.19***
6- HADS - Total	13.58	8.01	0.89					–	0.03	0.21***	0.14***
7- P RCOPE	17.87	4.53	0.85						–	0.19***	0.85***
8- N RCOPE	9.32	3.23	0.80							–	0.68***
9- Brief RCOPE- Total	27.19	6.04	0.81								–

*SD* Standard deviation, *α Cronbach’s α*, *CFS Physical fatigue* Chalder Fatigue Scale - Physical fatigue subscale, *CFS Mental fatigue* Chalder Fatigue Scale - Mental fatigue subscale, *CFS – Total* Chalder Fatigue Scale – Total score, *HADS – Depression* Hospital Anxiety and Depression Scale – Depression subscale, *HADS – Anxiety* Hospital Anxiety and Depression Scale – Anxiety subscale, *HADS – Total* Hospital Anxiety and Depression Scale – Total score, *P RCOPE* positive religious coping subscale, *N RCOPE* negative religious coping subscale, *Brief RCOPE- Total* Brief Religious Coping Scale – Total score

*p < 0.05, ***p < 0.001

Logistic regression analyses were conducted to examine the associations of sociodemographic factors, religious coping, mental fatigue, and physical fatigue with depression and anxiety under COVID-19 epidemic-related factors (Table 4). The results of single regression analysis confirm the significant association of most factors described by the chi-squared test with depression and anxiety (Table 2). Besides, it appears that depressive and anxious patients used more negative religious coping, while positive religious coping was only slightly associated with depression. The results showed also that mental fatigue and physical fatigue were associated significantly and positively with depression as well as with anxiety. The findings remained significant even after adjusted in the multiple logistic regression model. The latter indicated that physical fatigue was significantly associated with depression (aOR = 3.35, 95% CI: 2.51–4.50) and anxiety (aOR = 3.14, 95% CI: 2.37–4.18), and mental fatigue predicted significantly depression (aOR = 4.05, 95% CI: 2.91–5.71) and anxiety (aOR = 3.80, 95% CI: 2.78–5.20). Some sociodemographic variables remained significantly associated with depression and/or anxiety in multivariate logistic regression, including female gender, household income decline, tracking COVID-19 news, and having fear to contract this disease (aOR = 1.36 to 2.85). The model had a Nagelkerke’s R^2^ of 0.29, and 0.36 for depression and anxiety, respectively. This suggests that about 29.0% of the depression variance and 36.0% of the anxiety variance could be explained by this model, though this should be interpreted with caution, since “pseudo” R2 values are derived differently in logistic regression compared with linear regression (Table 4).

**Table 4:** Univariate and multivariate regression analysis of the association between religious coping, physical fatigue, mental fatigue, and sociodemographics with depression and anxiety

Variables	Depression		Anxiety	
	OR (95% CI)	aOR (95% CI)	OR (95% CI)	aOR (95% CI)
Age (Years)	0.97 (0.96–0.98)***	0.99 (0.97–1.02)	0.98 (0.97–0.99)***	1.00 (0.98–1.02)
Age stages (Years)				
≤ 45	1	1	1	1
> 45	0.55 (0.41–0.75)***	0.87 (0.50–1.51)	0.60 (0.43–0.82)***	0.70 (0.39–1.26)
Gender				
Male	1	1	1	1
Female	1.63 (1.31–2.04)***	1.36 (1.06–1.75)*	2.28 (1.81–2.85)***	2.04 (1.56–2.70)***
Status as a parent				
Having children	1	1	1	1
No children	1.36 (1.09–1.70)**	0.92 (0.67–1.27)	1.11 (0.89–1.39)	0.69 (0.49–0.98)*
Education				
Higher education	1	1	1	1
Primary or secondary education	1.00 (0.67–1.50)	1.18 (0.74–1.87)	1.11 (0.74–1.66)	1.17 (0.71–1.90)
Employment status				
Employed	1	1	1	1
Unemployed	1.05 (0.71–1.56)	1.20 (0.76–1.92)	1.29 (0.87–1.90)	1.47 (0.90–2.38)
Household income decline				
No	1	1	1	1
Yes	1.63 (1.28–2.08)***	1.40 (1.07–1.85)*	1.49 (1.17–1.88)***	1.33 (1.01–1.75)
Looked up regular news				
Seldom	1	1	1	1
Regularly	1.79 (1.23–2.63)***	1.66 (1.09–2.56)*	1.97 (1.31–3.03)***	1.58 (1.01–2.56)
Fear of COVID-19				
Low	1	1	1	1
High	2.24 (1.75–2.85)***	1.75 (1.35–2.32)***	3.28 (2.56–4.16)***	2.85 (2.17–3.70)***
CFS-Physical fatigue				
No fatigue	1	1	1	1
Severe fatigue	5.90 (4.55–7.72)***	3.35 (2.51–4.50)***	5.44 (4.28–6.96)***	3.14 (2.37–4.18)***
CFS-Mental fatigue				
No fatigue	1	1	1	1
Severe fatigue	7.69 (5.69–10.61)***	4.05 (2.91–5.71)***	6.82 (5.22–8.98)***	3.80 (2.78–5.20)***
P RCOPE	0.99 (0.96–1.01)	0.97 (0.95–1.00)	1.03 (1.01–1.06)**	1.01 (0.98–1.04)
N RCOPE	1.08 (1.05–1.12)***	1.05 (1.01–1.09)*	1.14 (1.10–1.18)***	1.11 (1.07–1.16)***
Nagelkerke R^2^ for the model	0.29			0.36

*OR* odds ratio, *aOR* adjusted odds ratio, *CI* confidence interval, *CFS- Physical fatigue* Chalder Fatigue Scale - Physical fatigue subscale, *CFS - Mental fatigue* Chalder Fatigue Scale - Mental fatigue subscale, *P RCOPE* Positive religious coping, *N RCOPE* Negative religious coping

*p < 0.05, **p < 0.01, ***p < 0.001

## Discussion

This study is a web-based survey aiming to examine the prevalence and associated variables of probable depressive and anxiety symptoms among 1435 respondents during the COVID-19 lockdown in Morocco. Therefore, the survey could have targeted only highly educated people having internet access. Unfortunately, this is one of the limitations of this study, because it is not representative of the general population.

The present findings demonstrated that nearly all respondents (*n* = 1301, 91.0%) showed a need to be regularly updated with the latest information about COVID 19. This can be explained by the fact that our survey was carried out in the early stage of the COVID-19 outbreak in Morocco.

Importantly, the results of this survey revealed that most of the surveyed subjects expressed a high level of compliance with quarantine (*n* = 1213, 85.0%), and protective measures (always washing hands, wearing a mask, and covering mouth when coughing and sneezing) (*n* = 979, 68.0%). These results can be partly explained by the fact that most respondents (*n* = 1332, 93.0%) were highly educated, and might be attributed to the efficiency of authorities’ awareness campaigns in the initial stage of the pandemic. Like few success stories [38], Morocco has adopted early quarantine measures, community mobilization, as well as large-scale testing of the population, which enabled the country to avoid a massive outbreak. Interestingly, the compliance with quarantine and protective measures was associated neither with depression nor with anxiety.

For concerns about COVID-19, 32.0% (*n* = 454) of the subject’s study expressed a high level of fear to contract COVID-19, and only 7.0% (*n* = 100) expressed a high level of fear to die from this disease. The fact that 91.0% (*n* = 1312) of participants had no chronic disease might explain why they were less afraid to contract COVID-19 and to die if infected.

Concerning the psychological impact of COVID-19 and quarantine measured by HADS, results revealed that 53.0% (*n* = 766) and 43.0% (*n* = 621) of the participants were respectively found to be depressed and anxious (HADS scores of 8 and above for both anxiety and depression). The elevated rates of depression and anxiety found in this study could be caused by other factors which were not measured in this study, mainly sleep disturbance. The latter was ascertained to be associated with anxiety, depression, and suicidal behavior [39]. Indeed, the prevalence of depression (14.6 to 48.3%) and anxiety symptoms (6.33 to 50.9%), stress disorder (7 to 53.8%), psychological distress (34.43 to 38%), and stress (8.1 to 81.9%) is higher during the COVID-19 outbreak than before in many countries [40]. In the United States, the depression prevalence was 3-fold higher during the COVID-19 outbreak than before [41], 32.7% of adults from the Chinese general population experienced elevated anxiety or depressive symptoms [42]. Moreover, nearly half of the Libyan population suffered from depressive symptoms, and one-fifth presented severe anxiety symptoms [43, 44].

Univariate logistic regression showed that depression and anxiety were associated with younger age and female gender, while multivariate logistic regression indicated that only female gender remained significant. Our results showed that depression and anxiety were more likely to occur in younger people age 45 years or younger. These findings are corroborated by a previous study in Morocco, which revealed that people aged less than 35 years present more depressive and anxiety symptoms than those aged 35 years or older [45]. Moreover, previous studies undertaken on the Chinese population have demonstrated that younger participants are more likely inclined to undergo unemployment, income decline, high frequency of access to pandemic news through social media, and prone to develop anxiety and depressive symptoms during COVID-19 outbreak than those aged above 40 years [42, 46, 47]. Concerning the female gender, its association with depression and anxiety during COVID-19 pandemic was found in several epidemiological studies [45, 48–50]. In Morocco, the prevalence of depression and anxiety among women is high compared to men [35, 36]. The differential risk of depression and anxiety may stem from biological sex differences, and COVID-19 related factors, such as lockdown, unemployment, and family violence [51, 52]. Previous studies have ascertained that violence against women leads to subsequent depression and anxiety [53, 54]. Accordingly, the rate of violence toward Moroccan women is estimated at around 54.4% [55], and it is susceptive to rise significantly under pandemic and resultant shutdowns [14, 49, 52, 53, 56]. In this regard, it is reasonable to hypothesize that violence against Moroccan women may be one stressor among others that are associated with the higher prevalence of depression and anxiety recorded during the COVID-19 outbreak. Of Note, other stressors such as chronic health issues, exercise, financial problems, excess information from mass media, and infection fears were reported to be associated with depression, anxiety, distress and life satisfaction [14, 57–60].

The present study demonstrated that the likelihood of having depression and/or anxiety was higher in participants reporting mental and/or physical fatigue. Indeed, fatigue and depression share specific symptoms such as sleep disturbances, poor concentration, and memory difficulties [43, 61]. This explains why patients with either fatigue or depression are reported to be two-fold prone to show an increased risk for the comorbid presentation of both traits, compared to the general population [62].

Fatigue, depression, and anxiety in the present study were negatively correlated with positive religious coping, and positively correlated with negative religious coping. These findings confirmed the widespread belief that religiosity provides a source of attitudes that can reframe negative events into less stressful frames [63]. Thereby, positive religious coping and the trust in God correlate with less stress and increased positive impact [64, 65], when negative religious coping is usually linked to undesirable physical and mental health indicators such as depression, anxiety, and fatigue [64–66]. The weak association found in this study between positive religious coping and depression, was reported in other studies which mentioned that in samples experiencing a specific challenge, the association between positive religious coping and depression is somewhat weaker or conflicting [67].

The results of this study should be interpreted in light of the following limitations. First, due to the limited resources available and the time-sensitivity of the COVID-19 outbreak, the snowball sampling strategy was adopted as a safe and the only option available during the total lockdown. The sampling was somehow biased since the questionnaire was administered to highly educated people who are the most internet users. Hence, these findings cannot, however, be extrapolated to all Moroccan populations. Second, this study did not collect contact details and personal information from the respondents mainly due to the ethical requirements on anonymity and confidentiality. As a result, we could not conduct a prospective study on the same group of participants in the future. Third, our findings were based on self-report data. Self-reported levels of depression, anxiety, and fatigue might be biased and may not always be aligned with assessment by health professionals. Notwithstanding the above limitations, this study provides invaluable information, from respondents across Morocco, about the initial psychological impact, fatigue, and religious coping associated with the spread of the COVID-19 and the lockdown on the Moroccan population.

## Conclusions

Overall, this study is one among few studies that have assessed the effects of the COVID-19 pandemic on depression, anxiety, fatigue, and religious coping among the Moroccan general population. It showed that the likelihood of having depression and anxiety was higher in participants reporting mental or physical fatigue and who had experienced negative religious coping. However, the likelihood of having depression and anxiety was lower in participants with positive religious coping. This highlights the importance of the development of psychological interventions to minimize psychological impact, anxiety, and depression, during the outbreak of COVID-19. Further research about the psychological impact of COVID − 19 and religious coping in Morocco is recommended especially to be compared to our work that was carried out at the beginning of the pandemic.

## Supplementary Information

**Additional file 1.**

## Data Availability

The datasets used and/or analysed during the current study are available from the corresponding author on reasonable request.

## Abbreviations

α: Cronbach’s α
Brief RCOPE: Brief Religious Coping Scale
CFS: Chalder Fatigue Scale
CFS - Mental fatigue: Chalder Fatigue Scale - Mental fatigue subscale
CFS- Physical fatigue: Chalder Fatigue Scale - Physical fatigue subscale
CI: Confidence interval
COVID-19: Coronavirus disease
HADS: Hospital Anxiety and Depression Scale – Total score
HADS – Anxiety: Hospital Anxiety and Depression Scale – Anxiety subscale
HADS – Depression: Hospital Anxiety and Depression Scale – Depression subscale
N RCOPE: Negative religious coping subscale
P RCOPE: Positive religious coping subscale
OR: Odds ratio
