# Supplementary material for: An investigation of the association between religious coping, fatigue, anxiety and depressive symptoms during the COVID-19 pandemic in Morocco: a web-based cross-sectional survey
Source: BMC Psychiatry. 2021 May 22;21:264. doi: 10.1186/s12888-021-03271-6 (PMC8140317; doi:10.1186/s12888-021-03271-6)
Supplement: Supplementary file 1 — Additional file 1. [file 12888_2021_3271_MOESM1_ESM.docx]

# An investigation of the association between religious coping, fatigue, anxiety and depressive symptoms during the COVID-19 pandemic in Morocco: A web-based cross-sectional survey

**Btissame ZARROUQ^1,2^, Nivine ABBAS^3^, Jaouad EL HILALY^4^, Achraf EL ASRI^1^, Samira ABBOUYI^1^, Majid OMARI^1,5^, Hicham MALKI^1^, Samira BOUAZZA^2^, Salma Ghofrane MOUTAWAKKIL^2^, Karima HALIM^5^,** [**Mohamed El Amine RAGALA**](https://www.sciencedirect.com/science/article/abs/pii/S0398762019302056#!)**^2,5^**.

^1^ Laboratory of Epidemiology and Research in Health Sciences, Faculty of Medicine and Pharmacy, Sidi Mohamed Ben Abdellah University, Fez, Morocco.

^2^ Teachers Training College (Ecole Normale Superieure), Department of Biology and Geology, Sidi Mohamed Ben Abdellah University, Fez, Morocco.

^3^ Public Health Department, Faculty of Health Sciences, University of Balamand, Lebanon.

^4^ Laboratory of Pedagogical and Didactic Engineering of Sciences and Mathematics, Regional Center of Education and Training (CRMEF) of Fez. Morocco.

^5^ Laboratory of Natural Substances, Pharmacology, Environment, Modeling, Health & Quality of Life, Faculty of Sciences Dhar El Mahraz, Sidi Mohamed Ben Abdellah University, Fez, Morocco.

**Informed consent**

# An investigation of the association between religious coping, fatigue, anxiety and depressive symptoms during the COVID-19 pandemic in Morocco: A web-based cross-sectional survey

**Introduction**

Morocco has been affected by the COVID-19 pandemic, similar to other countries in the world. The questionnaire, developed by professors from Sidi Mohamed Ben Abdellah University in Fez, aims to measure the effects of the COVID-19 pandemic on the psychological impact, fatigue, and religious coping among the Moroccan general population.

You have been chosen randomly, and your participation remains voluntary.

We are going to give you information about the research below. We invite you to be part of this research. There are no correct and wrong answers. Each question has different possibilities to answer. You are asked to choose one of these answers by marking (×) within the box that describes your true feeling.

Before answering the survey questions please read the "Informed Consent" document and confirm your consent to participate in this research.

**What is the purpose of this research?**

The purpose of this research is to explore the psychological and social impact of the COVID-19 pandemic on your life and the factors that help promote resilience to face the threat of this pandemic. This research needs to gather accurate information from all kinds of people affected by the spread of this virus in Morocco. By conducting this study, we learn about the psychological effects of the quarantine and the pandemic on Moroccan citizens. Also, this research explores the association, if any, between religious coping and the psychological state of the citizens during these difficult circumstances.

The information gained from the results of this research will guide future efforts and will help in developing prevention and treatment strategies to enhance resilience and reduce stress and its negative effects on the health of the Moroccan people.

**Why am I invited to participate in this research study?**

We ask you to participate in this research study because you have indicated in your social media profile that you are an adult (18+) or because someone else shared the link to the search with you as an adult interested in this survey.

**What should I know about the research study?**

You can ask all the questions you want before you decide. Data will be collected through an online questionnaire at one point in time and no follow up of participants will be required. **How long does it take to complete the survey?**

We expect you to complete this online search survey in 10-15 minutes.

**What should I do to participate?**

If you accept to participate, you will need to fill in the online questionnaire. You'll be asked questions about how the spread of COVID-19 affects your life and family life, your experience of depression, anxiety, uncertainty, and your concerns about the future.

**Any risks for my participation in this study?**

By participating in this research there are no risks anticipated for the participants. We will ask you questions about life, including experiences that can be stressful. You may find some sensitive questions, or you may feel some discomfort when thinking about previous experiences. We will ask if you have been in financial distress with the CORONA virus and if you have lost a loved one because of this pandemic.

If you would like information or support for you or a family member regarding mental health, please visit the Sidi Mohamed Ben Abdellah University website and see the phone numbers assigned to the remote psychological counseling service.

No one in this search will be able to identify you or your answers, and no one will know if you participated in the study. Therefore, there is no risk of being involved in this research.

**Is my participation voluntary?**

Your participation in this research is entirely voluntary. It is your choice whether to participate or not.

**What happens if I don't want to be in this search?**

You have the right to refuse. You do not have to take part in this research if you do not wish to do so. It is your choice and all of your rights will still be respected. Your choice not to participate in this study will not adversely affect your right to any current or future medical care, academic status as a student, or your current or future job.

**Is the information collected kept confidential?**

This survey is completely anonymous. We do not collect any identifying information. For example, we will not request your name or contact information, nor will we collect IP addresses. The information that we collect from this research project will be kept confidential. Anonymous data will be stored on encrypted and password-protected computers used by the research team supervising the study. All the information collected will be used only for scientific reasons.

**Does my participation in this study help me in any way?**

If you participate in this research, there may not be any benefit for you directly. However, your participation is likely to help us find the answer to the research question. Society and future generations are likely to benefit.

**How many people will be included in this study?**

We expect thousands of people to participate in this study.

**What are my responsibilities if I participate in this research?**

If you participate in this research, you will be responsible for providing honest answers to your experiences and behaviors.

**Will it cost me anything to participate in this research study?**

Participating in this research study will not result in any costs for you.

**Will I be compensated for my participation?**

You will not be given any compensation for your participating in this research.

**Will I receive the results of the research?**

Many questionnaires conducted in research studies are only done for research and have no clear meaning for health care. Researchers will not contact you or share your results. The results of this research will be published for interested people to learn from our research.

**Who to Contact?**

To ask questions about this research study, research results , or other interests now or later, even after the study has started, please contact Pr. Btissame Zarrouq, professor at Sidi Mohamed Ben Abdellah University in Fez, and a researcher in the Laboratory of Epidemiology and Research in Health Sciences at the Faculty of Medicine and Pharmacy in Fez.

E-mail address: btissame.zarrouq@usmba.ac.ma

**This proposal has been reviewed and approved by the hospital-university ethics committee of Sidi Maohamed Ben Abdellah University (N° 09/20).**

**After you confirm your approval to participate in the search, you can start answering the questionnaire. Thank you.**

**Online Questionnaire**

# An investigation of the association between religious coping, fatigue, anxiety and depressive symptoms during the COVID-19 pandemic in Morocco: A web-based cross-sectional survey

- **Socio-demographic data**

1. What is your sex?

❒ Male

❒ Female

1. How old are you? …….. Years
2. What is your marital status?

❒ Single

❒ Married

❒ Divorced

❒ Widowed

1. What is your parental status?

❒ I have children

❒ I don’t have children

1. What is your educational level?

❒ Primary

❒ Secondary

❒ University

1. What is your employment status?

❒ Unemployed

❒ Student

❒ Employed in the public sector

❒ Employed in the private sector

❒ Self-employed

❒ Retired

1. What are your living arrangements?

❒ Living with family

❒ Living with a roommate

❒ Living alone

1. What is your location of residence?

❒ Urban

❒ Rural

- **Knowledge and adherence to quarantine and infection control directives**

1. Do you need regular updates for the latest information about the COVID-19 infection (route of transmission, the availability of medicines, the number of infected cases and death, advice on prevention of the COVID-19)?

❒ Yes

❒ No

1. What is your level of compliance with quarantine measures?

❒ Low compliance

❒ Average compliance

❒ High compliance

1. What is your level of compliance with infection control directives (always washing hands, wearing a mask, and covering mouth when coughing and sneezing)?

❒ Low compliance

❒ Average compliance

❒ High compliance

- **Concerns about COVID-19 :**

1. To what level do you fear contracting COVID-19 during the current outbreak?

❒ Low

❒ Middle

❒ High

1. To what level do you fear to die if infected with COVID-19?

❒ Low

❒ Middle

❒ High

1. Are you suffering from a chronic disease (diabetes, hypertension, for example)?

❒ Yes

❒ No

1. Are you satisfied with your life during the epidemic of COVID-19?

❒ Yes

❒ No

1. Are you experiencing financial problems (household income declined) because of the COVID-19 epidemic?

❒ Yes

❒ No

1. Do you know someone died from COVID-19?

❒ Yes

❒ No

- **The psychological impact of COVID-19 and quarantine**

**(**Hospital Anxiety and Depression Scale (HADS))

The following questions explore the level of depression or anxiety you have, if any, within the past two weeks in which Morocco has suffered from the spread of the COVID-19 epidemic.

It is not necessary to think too much to choose the answer, but rather to prefer spontaneous answers.

| 1. I feel tense or 'wound up':  - Most of the time - A lot of the time - From time to time, occasionally - Not at all | 1. I feel as if I am slowed down:  - Nearly all the time - Very often - Sometimes - Not at all |
| --- | --- |
| 1. I still enjoy the things I used to enjoy:  - Definitely as much - Not quite so much - Only a little - Hardly at all | 1. I get a sort of frightened feeling like 'butterflies' in the stomach:  - Not at all - Occasionally - Quite Often - Very Often |
| 1. I get a sort of frightened feeling as if something awful is about to happen:  - Very definitely and quite badly - Yes, but not too badly - A little, but it doesn't worry me - Not at all | 1. I have lost interest in my appearance:  - Definitely - I don't take as much care as I should - I may not take quite as much care - I take just as much care as ever |
| 1. I can laugh and see the funny side of things:  - As much as I always could - Not quite so much now - Definitely not so much now - Not at all | 1. I feel restless as I have to be on the move:  - Very much indeed - Quite a lot - Not very much - Not at all |
| 1. Worrying thoughts go through my mind:  - A great deal of the time - A lot of the time - From time to time, but not too often - Only occasionally | 1. I look forward with enjoyment to things:  - As much as I ever did - Rather less than I used to - Definitely less than I used to - Hardly at all |
| 1. I feel cheerful:  - Not at all - Not often - Sometimes - Most of the time | 1. I get sudden feelings of panic:  - Very often indeed - Quite often - Not very often - Not at all |
| 1. I can sit at ease and feel relaxed:  - Definitely - Usually - Not Often - Not at all | 1. I can enjoy a good book or radio or TV program:  - Often - Sometimes - Not often - Very seldom |

- **The physical and mental symptoms variables related to COVID-19 and quarantine**

**(**the Chalder Fatigue Scale **(**CFS))

The following questions try to measure your physical and mental fatigue during the past two weeks in which Morocco has suffered from the spread of the COVID-19 epidemic.

|  | Less than usual | No more than usual | More than usual | Much more than usual |
| --- | --- | --- | --- | --- |
| 1. Do you have problems with tiredness? |  |  |  |  |
| 1. Do you need to rest more? |  |  |  |  |
| 1. Do you feel [sleepy](https://me-pedia.org/wiki/Somnolence) or drowsy? |  |  |  |  |
| 1. Do you have problems starting things? |  |  |  |  |
| 1. Do you have lack of energy? |  |  |  |  |
| 1. Do you have [less strength in your muscles](https://me-pedia.org/wiki/Muscle_fatigability)? |  |  |  |  |
| 1. Do you feel weak? |  |  |  |  |
| 1. Do you have [difficulties concentrating](https://me-pedia.org/wiki/Cognitive_dysfunction)? |  |  |  |  |
| 1. Do you make [slips of the tongue when speaking](https://me-pedia.org/wiki/Speech_difficulties)? |  |  |  |  |
| 1. Do you find it more [difficult to find the right word](https://me-pedia.org/wiki/Word-finding_problems)? |  |  |  |  |
|  | Better than usual | No worse than usual | Worse than usual | Much worse than usual |
| 1. How is your [memory](https://me-pedia.org/wiki/Memory_problems)? |  |  |  |  |

- **Religious coping (**The Brief Religious Coping Scale (Brief RCOPE))

Try to think about your relationship with GOD almighty during the past two weeks under the COVID-d 19 pandemic and answer the following questions:

|  | Not at all | Some-what | Quite a bit | A great deal |
| --- | --- | --- | --- | --- |
| 1. Looked for a stronger connection with God through my fast and my prayers. |  |  |  |  |
| 1. Sought after God’s mercy, care and protection. |  |  |  |  |
| 1. Sought to do good deeds to receive God’s satisfaction. |  |  |  |  |
| 1. Tried to ease my problems by following the teachings of the religion to which I belong. |  |  |  |  |
| 1. Asked God to stand with me and strengthen me in troubled times. |  |  |  |  |
| 1. Asked God for forgiveness for my sins and actions that might anger God. |  |  |  |  |
| 1. Focused on religion to stop worrying about my problems. |  |  |  |  |
| 1. Wondered whether God had abandoned me. |  |  |  |  |
| 1. Felt punished by God for my lack of devotion (fasting and prayer). |  |  |  |  |
| 1. Wondered what I did for God to punish me. |  |  |  |  |
| 1. Questioned God’s love for me. |  |  |  |  |
| 1. Wondered whether people of faith had abandoned me. |  |  |  |  |
| 1. Decided the devil made this happen. |  |  |  |  |
| 1. Questioned the power of God. |  |  |  |  |

**References**

- **The psychometric scales used in this questionnaire were taken from:**
- Zigmond AS, Snaith RP. The hospital anxiety and depression scale. Acta Psychiatr Scand. 1983 Jun;67(6):361–70.
- Chalder T, Berelowitz G, Pawlikowska T, Watts L, Wessely S, Wright D, et al. Development of a fatigue scale. J Psychosom Res. 1993 Feb 1;37(2):147–53.
- Pargament KI, Smith BW, Koenig HG, Perez L. Patterns of positive and negative religious coping with major life stressors. J Sci Study Relig. 1998;37(4):710–24.
- **The validated Arabic version of the psychometric scales used in this questionnaire was taken from:**
- Terkawi AS, Tsang S, AlKahtani GJ, Al-Mousa SH, Musaed SA, AlZoraigi US, et al. Development and validation of Arabic version of the Hospital Anxiety and Depression Scale. Saudi J Anaesth. 2017 Jan 5;11(5):11.
- Mcllvenny, S. The translation into arabic and revalidation of a fatigue questionnaire. Eastern Mediterranean Health Journal. 1999;5 (3), 503–14.
- Al-Hadethe A, Hunt N, Thomas S, Al-Qaysi A. Cross-Cultural Validation and Psychometric Properties of the Arabic Brief Religious Coping Scale (A-BRCS). J Relig Health. 2016 Feb 1;55(1):16–25.
